# Long-term exposure to particulate air pollution and brachial artery flow-mediated dilation in the Old Order Amish

**DOI:** 10.1186/s12940-020-00593-y

**Published:** 2020-05-14

**Authors:** Shabnam Salimi, Jeff D. Yanosky, Dina Huang, Jessica Montressor-Lopez, Robert Vogel, Robert M. Reed, Braxton D. Mitchell, Robin C. Puett

**Affiliations:** 1 Department of Medicine, University of Maryland School of Medicine, Baltimore, MD USA; 2 Department of Public Health Sciences, College of Medicine, The Pennsylvania State University College of Medicine, 90 Hope Drive, Hershey, PA 17033 USA; 3 Department of Epidemiology and Biostatistics, University of Maryland School of Public Health, College Park, MD USA; 4 Maryland Institute of Applied Environmental Health, University of Maryland School of Public Health, College Park, MD USA; 5 Department of Medicine, Division of Pulmonary and Critical Care Medicine, University of Maryland School of Medicine, Baltimore, MD USA; 6 Geriatrics Research and Education Clinical Center, Baltimore Veterans Administration Medical Center, Baltimore, MD USA

**Keywords:** Endothelial function, Cardiovascular disease, Air pollution, Particulate matter

## Abstract

**Background:**

Atmospheric particulate matter (PM) has been associated with endothelial dysfunction, an early marker of cardiovascular risk. Our aim was to extend this research to a genetically homogenous, geographically stable rural population using location-specific moving-average air pollution exposure estimates indexed to the date of endothelial function measurement.

**Methods:**

We measured endothelial function using brachial artery flow-mediated dilation (FMD) in 615 community-dwelling healthy Amish participants. Exposures to PM < 2.5 μm (PM_2.5_) and PM < 10 μm (PM_10_) were estimated at participants’ residential addresses using previously developed geographic information system-based spatio-temporal models and normalized. Associations between PM exposures and FMD were evaluated using linear mixed-effects regression models, and polynomial distributed lag (PDL) models followed by Bayesian model averaging (BMA) were used to assess response to delayed effects occurring across multiple months.

**Results:**

Exposure to PM_10_ was consistently inversely associated with FMD, with the strongest (most negative) association for a 12-month moving average (− 0.09; 95% CI: − 0.15, − 0.03). Associations with PM_2.5_ were also strongest for a 12-month moving average but were weaker than for PM_10_ (− 0.07; 95% CI: − 0.13, − 0.09). Associations of PM_2.5_ and PM_10_ with FMD were somewhat stronger in men than in women, particularly for PM_10_.

**Conclusions:**

Using location-specific moving-average air pollution exposure estimates, we have shown that 12-month moving-average estimates of PM_2.5_ and PM_10_ exposure are associated with impaired endothelial function in a rural population.

## Introduction

Endothelial cells residing in the inner layer of blood vessels are a key determinant of vascular health. Endothelial cell function includes vasoconstriction and vasodilation through nitric oxide release. Endothelial damage results in aggregation of platelets and their adhesion to the vascular wall. These processes can lead to thromboses resulting in cardiovascular events (e.g., stoke, incident myocardial infarction, angina, coronary revascularization, cardiac arrest, cardiovascular (CVD)-associated death [25];); such thrombotic conditions can be addressed medically to prevent cardiovascular events in patients with and without known CVD [36]. Endothelial dysfunction is widely recognized as an initial and reversible precursor in the progression of atherogenesis [2]. Through continued oxidation and inflammation, factors contributing to endothelial dysfunction lead to the progression of atherosclerosis.

Brachial artery endothelial function is affected by several established risk factors for CVD, including hypertension, homocystinuria, oxidized low density lipoprotein (LDL) cholesterol, tobacco smoking, and oxidative stress [6, 7]. Further, brachial artery flow-mediated dilation (FMD), assessed non-invasively with ultrasound, is a broadly used assessment of endothelial function in adults [6, 12, 15, 32, 36].

A substantial body of epidemiologic evidence has linked exposure to particulate matter (PM) air pollution to a wide array of adverse cardiovascular outcomes [1, 3, 4, 10, 13, 17, 19–21, 30, 34], including some effects considered to be directly downstream of impaired endothelial function. For example, exposure to PM < 2.5 μm in aerodynamic diameter (PM_2.5_) has been associated with cardiovascular risk factors including hypertension, systemic inflammation, oxidative stress, and established atherosclerosis [4, 31]. Though results from some earlier studies were inconsistent ([4] and references therein), two recent epidemiologic studies that used PM air pollution exposure modeling similar to that in the present analysis have reported associations between long-term PM_2.5_ exposure and brachial artery FMD [13, 34]. Wilker et al. used a 1-year average of spatio-temporal PM_2.5_ model predictions from the year 2001, with 50 m spatial resolution, as a surrogate for long-term exposure. Krishnan et al. applied a hierarchical spatio-temporal universal kriging model of two-week average PM_2.5_ levels, taking a 1-year average of model predictions for the year 2000. Neither of these analyses used moving averages specific to the date of FMD measurement, nor did they report on whether results were sensitive to the selected averaging period of 1 year as compared to shorter or longer averaging times (with the exception that Krishnan et al. evaluated 1 and 2 days prior to FMD measurement). Also, neither of these analyses was performed in a primarily rural population. We sought to address these gaps using a genetically homogenous, geographically stable population. We hypothesized that elevated PM exposures result in detrimental effects on endothelial function assessed by FMD. Our objective was to determine whether and over what averaging period long-term exposure to PM_2.5_ and PM < 10 μm in aerodynamic diameter (PM_10_) were associated with FMD in a community-based sample of 615 healthy individuals from an Amish community in Lancaster County, PA.

## Methods

### Study design

We performed a retrospective cohort study of participants recruited into the Heredity and Phenotype Intervention (HAPI) Heart Study [16]. The HAPI Heart Study was conducted among the Amish community in Lancaster County, PA and designed to identify potential genetic and environmental risk factors of CVD [16]. Relevant to the present study, the Lancaster Amish are characterized by high levels of physical activity, homogeneity of socioeconomic status and lifestyle, limited use of prescription medications, and relative geographic stability. Further details of the HAPI Heart Study are available elsewhere [16]. Briefly, the HAPI Heart Study was a community-wide study of clinically healthy individuals aged 20 years and older with the following major exclusions: currently pregnant or < 6 months post-partum, blood pressure at the time of screening > 180/105 mmHg, and unable or unwilling to safely discontinue medications potentially affecting study outcomes (prescription medication use has been documented to be lower among this population than the general population [24]). A total of 868 participants were enrolled in the HAPI Study from 2003 to 2006, of whom endothelial function was assessed on the exam day by FMD in 615 participants (some of whom in family groups) in the present analysis.

Demographic, health behavior (e.g., smoking and eating habits), and family and medical history information was obtained through interviews by a study nurse and Amish liaison in participant homes. Body mass index (BMI) and FMD were measured at the Amish Research Clinic in Lancaster County, PA. All study participants provided written informed consent prior to data collection, and the HAPI Heart Study protocol was approved by the Institutional Review Board at the University of Maryland. The protocol for the present air pollution ancillary study was approved by the Institutional Review Boards at the University of Maryland and Pennsylvania State University.

### Brachial artery FMD

Endothelial function was measured by brachial artery reactivity test (BART) to assess FMD using standardized procedures based on the International Brachial Artery Task Force and on expert guidelines [5, 9, 33]. All participants were fasting overnight (8–12 h) and were abstinent from food, caffeine, alcohol, and smoking. All medications (vasoactive and other), vitamins, and supplements were discontinued for 7 days prior to the study.

Base brachial artery diameter (BAD) and blood velocity were measured in the left brachial artery above the antecubital fossa using 11 mHz ultrasound (Phillips HDI 5000CV) with participants sitting supine for 15 min prior to the measurement. Then, using a standard sphygmomanometer cuff above the antecubital fossa, inflation was applied by 20 mmHg above systolic blood pressure for 5 min to occlude blood flow to the brachial artery and induce ischemia. Following the fast deflation of the cuff to induce post-ischemic hyperemia, the blood velocity and brachial artery diameter were recorded.

All images were measured in a blinded fashion by a trained technician and manually analyzed by a cardiologist as a single reader of the records. Percent FMD was computed as: $\left(\frac{MaxAD- BAD}{BAD}\right)\ast 100\%$, where *MaxAD* is the maximum brachial artery diameter.

### Shear stress and response to shear stress

Vascular endothelial cells are exposed to blood velocity-mediated shear stress. Shear stress is the product of blood viscosity by shear rate:

$$ Shear\, \, stress = \eta\frac{\text{V}}{\text{r}}$$

η = Blood viscosity; V = Blood velocity; r = Brachial artery diameter.

Assuming blood viscosity is constant, an increase in blood velocity leads to an increase in wall shear stress, which then evokes endothelial cell-mediated nitric oxide release and vessel dilation to decrease the shear stress. In the context of endothelial dysfunction, however, dilation following increased blood flow is impaired and response to shear stress is low [11].

After brachial artery occlusion, blood velocity increases which results in shear stress:

$$ Shear\ stress\sim \frac{Post\ occlusion\ blood\ velocity}{BAD} $$

The vasodilation (*MaxAD*) following cuff deflation and hyperemia occurs as the response to the shear stress [11]:

$$ Response\ to\ Shear\ Stress\sim \frac{Post\ occlusion\ blood\ velocity}{MaxAD} $$

The units of shear stress and response to shear stress were cm S^− 1^ mm^− 1^.

### Particulate air pollution exposure assessment

Residential addresses collected at the time of interview were geocoded using ArcGIS 9.3 software (ESRI, Redlands, California). Long-term exposures to PM_2.5_ and PM_10_ were estimated by applying previously-developed GIS-based spatio-temporal generalized additive mixed models (GAMMs) of PM_2.5_ and PM_10_ monthly-average mass concentrations, respectively [18, 35], at the participant’s geocoded residential addresses. These spatio-temporal models included 1) monthly spatial smooth terms and 2) smooth regression terms of a) GIS-based covariates and b) time-varying meteorological covariates. The models were developed using PM_2.5_ and PM_10_ monitoring data collected across the conterminous US from 1999 to 2011 (2018 and 2044 monitoring sites, respectively). Exposure models were validated using cross-validation and had high predictive performance (cross-validation R^2^’s of 0.77 and 0.58 for PM_2.5_ and PM_10_, respectively). Monthly exposure estimates specific to the residential location of each participant were averaged over a 12-month time period prior to the date of FMD measurement (the month prior to FMD measurement and the 11 months before that) for use in our *a priori* analysis. Also, in subsequent analyses, moving averages over time periods of 1, 6, 24, 36, 60, and 84 months were generated. Monthly exposure estimates were used to construct sets of polynomial distributed lag (PDL) basis coefficients [26].

### Statistical analysis

Linear mixed-effect regression models were used to evaluate associations between PM_2.5_ and PM_10_ exposure metrics with endothelial function measured by brachial artery FMD. Analyses were conducted using SAS version 9.4 (Cary, North Carolina) and Mixed Models Analysis for Pedigrees (MMAP website [14, 29]); mixed-effect models included a random effect for family structure to allow for the relatedness of participants within the Amish community. Statistical tests were 2-sided and *p*-values< 0.05 were considered statistically significant. In our regression models, we evaluated age, BMI, BAD, sex, age by sex, smoking (current, ever, never), season of the year (four seasons based on calendar month), serum cholesterol, serum triglyceride, and hypertension as potential confounders. We repeated the above analyses excluding BAD from the model. We also performed the above analyses stratified by age (younger than 50 vs. 50 years and older) by sex. Using interaction terms to assess effect modification, we evaluated: 1) age by sex 2) PM_2.5_ or PM_10_ by age, 3) PM_2.5_ or PM_10_ by sex, and 4) PM_2.5_ or PM_10_ by sex and age < 50 using interaction terms. We did not evaluate PM_2.5_ or PM_10_ by smoking interactions because none of our female participants were ever-smokers.

Because shear stress induces release of nitric oxide from endothelial cells resulting in response to shear stress, we evaluated associations of PM_2.5_ and PM_10_ with shear stress and response to shear stress with adjustment for BAD and the other covariates mentioned above.

As *a priori* hypotheses, we first fit models using 12-month moving-averages of PM_2.5_ and PM_10_ exposures. Next, in subsequent exploratory analyses, we evaluated other averaging periods (1, 6, 24, 36, 60, 84 months). The steps in our model development process were as follows: First, we fit crude models for a given averaging period for PM_2.5_ and PM_10_, then added potential confounders to these models, and next evaluated effect modification.

To evaluate the assumption of a constant effect (*i.e.,* one that does not taper off in time) of PM_2.5_ or PM_10_ exposure over a given averaging period, we performed PDL models followed by Bayesian model averaging (BMA) [27]. In exploratory analyses, we first identified the averaging period with the largest absolute value of effect among the fully adjusted moving-average models. We then fit PDL models from 0 to 5th order using the number of months in the averaging period selected above as the lag period. We then used BMA to calculate the probability-weighted average of the coefficients, given the data, from the resulting 6 PDL models. All final models were performed using linear mixed-effects models to account for family structure among participants (referred to as “polygenic mixed-effects models”).

To compare the strength of associations of endothelial function measures between PM_2.5_ and PM_10_, we normalized the PM exposure variables by subtracting their respective mean and dividing by their respective standard deviation. All results presented are based on normalized exposures, except those in Table S3.

## Results

### Study population

**Fig. 1:**
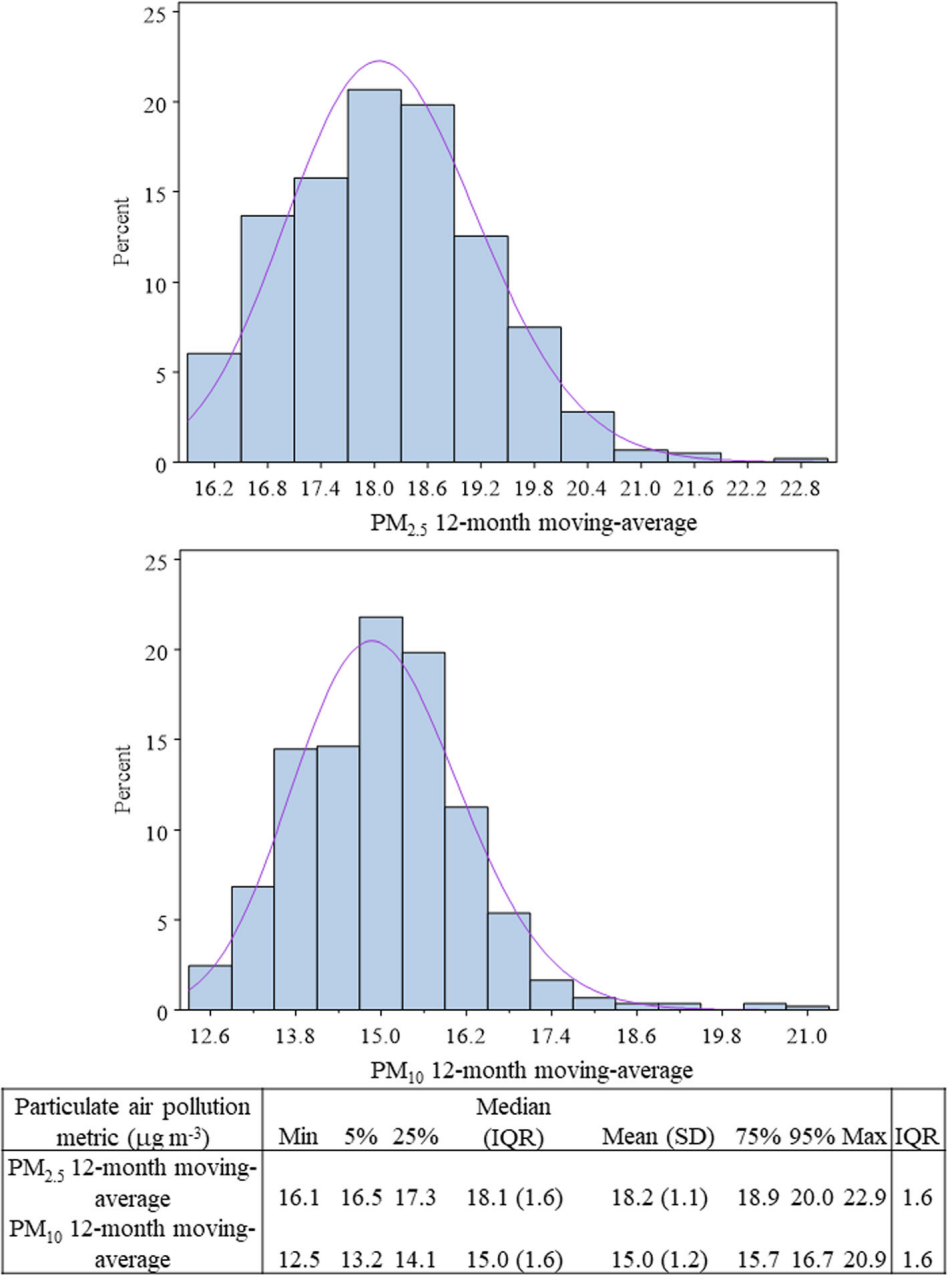
Distributions and summary statistics of the 12-month moving average PM_2.5_ and PM_10_ exposures, prior to normalization

Participant characteristics are presented in Table 1. The mean age in our study population was 43.5 years (SD: 13.9) and 43.7% were women. Among men, 20.5% were current smokers and 25.7% were ever smokers; in contrast none of the women reported smoking. Few participants reported a history of hypertension (3.7%), high cholesterol (16.8%), diabetes mellitus (0.8%), or heart attack (1.0%). FMD measures were approximately normally distributed with a mean value of 10.5% (SD: 5.8). Mean PM_2.5_ and PM_10_ 12-month moving averages were 18.2 μg m^− 3^ (SD: 1.1; interquartile range (IQR): 1.6) and 15.0 μg m^− 3^ (SD: 1.2; IQR: 1.6), respectively. Distributions of the PM_2.5_ and PM_10_ exposure metrics and additional summary statistics are shown in Fig. 1.

**Table 1:** Characteristics of study participants

Clinical characteristic	Mean (SD) or N (%)		
	*Across all*	*Men*	*Women*
Number of participants	615 (100%)	346 (56.3%)	269 (43.7%)
Age at examination (years)	43.5 (13.9)	42.5 (13.7)	44.9 (14.1)
Current smokers	71 (11.5%)	71 (20.5%)	0 (0%)
Ever smokers (not currently smoking)	89 (14.4%)	89 (25.7%)	0 (0%)
Never smokers	455 (74.0%)	186 (53.8%)	269 (100%)
Body mass index (kg m^−2^)	26.3(4.1)	25.5 (3.2)	27.3 (4.8)
Hypertension (diagnosed)	82 (13.3%)	42 (12.4%)	40 (14.9%)
High cholesterol (self-report)	103 (16.8%)	53 (15.3%)	50 (18.6%)
Diabetes mellitus (self-report)	5 (0.8%)	3 (0.9%)	2 (0.7%)
Myocardial infarction (self-report)	6 (1.0%)	4 (1.2%)	2 (0.7%)
Vascular measures			
Baseline brachial artery diameter (BAD; mm)	3.7 (0.7)	4.1 (0.4)	3.1 (0.4)
Flow-mediated dilation (FMD; %)	10.5 (5.8)	8.4 (4.9)	13.2 (5.9)
Shear stress (cm S^−1^ mm^−1^)	22.2 (7.5)	20.5 (6.1)	24.4 (8.6)
Pre-occlusive blood velocity (cm S^−1^)	7.8 (6.5)	9.5 (6.7)	5.6 (5.5)
Post-occlusive blood velocity (cm S^−1^)	79.5 (23.4)	82.9 (21.5)	74.9 (25.3)
Response to shear stress (cm S^−1^ mm^− 1^)	20 (6.6)	18.9 (5.5)	21.5 (7.5)

### Associations of PM_2.5_ and PM_10_ with FMD and shear stress measures

Table 2 shows associations of PM_2.5_ and PM_10_ exposure with FMD across all participants and for men and women separately with effect sizes for a normalized change in PM exposure (calculated by subtracting the mean and dividing by the SD). To afford comparisons with other studies, we also present these associations for a 10 μg m^− 3^ increment in PM_2.5_ or PM_10_ exposure in Table S3. Regression models were adjusted for age, BMI, and BAD as linear variables, and sex, smoking (current, ever, never), season of the year (four seasons based on calendar month), and hypertension as categorical variables. For neither PM_2.5_ nor PM_10_ was there significant evidence for a PM by sex interaction (*p* > 0.10).

**Table 2:** Associations of PM_2.5_ and PM_10_ exposure metrics and FMD (%), across all participants and by sex, for increases in normalized PM_2.5_ or PM_10_ exposure, in fully adjusted models

Particulate air pollution metrics (normalized)	*Across all*				*Men*				*Women*			
	β	SE	*p*-value	95% CI	β	SE	*p*-value	95% CI	β	SE	*p*-value	95% CI
PM_2.5_ 12-month moving-average*	−0.07	0.03	0.03	−0.13, − 0.09	−0.09	0.04	0.04	− 0.16, − 0.01	−0.07	0.05	0.2	−0.17, 0.03
PM_10_ 12-month moving-average*	−0.09	0.03	0.007	−0.15, − 0.03	−0.16	0.05	0.001	−0.26, − 0.06	−0.06	0.05	0.2	−0.16, 0.04

*All models adjusted for age, sex, age by sex interaction, smoking (except in models for women only because there were no ever smokers), BMI, season, year, hypertension, and base brachial artery diameter

*PM*_*2.5*_*.* Associations for PM_2.5_ were strongest (*i.e.,* furthest from zero), among the averaging periods considered, for a 12-month moving average. There was a significant inverse association between long-term exposure to PM_2.5_ and FMD: For a one unit increase in normalized 12-month moving-average PM_2.5,_ FMD decreased by 0.07 (95% CI: − 0.13, − 0.09; *p* = 0.03) in our polygenic mixed-effects model. We repeated the analyses excluding BAD from the model and the effect sizes were identical.

In sex-stratified analyses, PM_2.5_ was significantly associated with FMD in men (β = − 0.09; 95% CI: − 0.16, − 0.01; *p* = 0.04), but not in women (β = − 0.07; 95% CI: − 0.17, 0.03; *p* = 0.2) (Table 2). We also performed sex and age-stratified analyses: Associations remained moderately larger in men than in women and were larger in older (≥ 50 years) as compared to younger (< 50 years) individuals, though none were statistically significant in these subgroups (Table S2).

PM_2.5_ was not significantly associated with response to shear stress in the total population (*p* > 0.4), or in sub-analyses of men only or women only (*p* > 0.3 for both) (Table 3).

**Table 3:** Associations of PM_2.5_ and PM_10_ exposure metrics and response to shear stress (cm S^−1^ mm^− 1^), across all participants and by sex, for increases in normalized PM_2.5_ or PM_10_, in fully adjusted models

Particulate air pollution metrics (normalized)	*Across all*				*Men*				*Women*			
	β	SE	*p*-value	95% CI	β	SE	*p*-value	95% CI	β	SE	*p*-value	95% CI
PM_2.5_ 12-month moving-average*	0.03	0.03	0.4	−0.04, 0.11	− 0.02	0.04	0.7	−0.10, 0.06	0.07	0.06	0.3	−0.05, 0.19
PM_10_ 12-month moving-average*	0.07	0.04	0.08	−0.01, 0.14	0.01	0.05	0.8	−0.08, 0.10	0.11	0.06	0.08	−0.01, 0.22

*All models adjusted for age, sex, age by sex interaction, smoking (except in models for women only because there were no ever smokers), BMI, season, year, hypertension, and base brachial artery diameter

*PM*_*10*_*.* As for PM_2.5_, associations for PM_10_ were strongest (*i.e*., furthest from zero), among the averaging periods considered, for a 12-month moving average. There was a significant inverse association between long-term exposure to PM_10_ and FMD. This association was stronger than that for PM_2.5_ for the equivalent averaging period. For a one unit increase in normalized 12-month moving-average PM_10_, FMD decreased by 0.09 (95% CI: − 0.15, − 0.03; *p* = 0.007) in our polygenic mixed-effects model. There was no evidence of effect modification in associations between 12-month moving-average PM_10_ and FMD by age or sex (*p* > 0.05 for each). However, the association between FMD and PM_10_ was stronger and more significant in men (β = − 0.16; 95% CI: − 0.26, − 0.06; *p* = 0.001) than in women (β = − 0.06, 95% CI: − 0.16, 0.04; *p* = 0.2) (Table 2).

In age- and sex-stratified analyses, PM_10_ was more strongly associated with FMD in men than in women in both younger and older individuals. Only in the subgroup of men younger than age 50 years did the association between PM_10_ and FMD achieve statistical significance (β = − 0.16; 95%CI: − 0.25, − 0.04; *p* = 0.005) (Table S2).

PM_10_ was marginally associated with response to shear stress (β = 0.07; 95% CI: − 0.01, 0.14; *p* = 0.08) in the total study population, and in sex-stratified analyses the effect size was considerably larger in women compared to men, although in neither sex did the association achieve statistical significance (β = 0.11; *p* = 0.08 in women and β = 0.01; *p* = 0.8 in men) (Table 3).

### Time course of effect of PM_2.5_ and PM_10_ on FMD

Because associations of PM_2.5_ and PM_10_ moving averages were strongest for the time period 12 months prior to the date of FMD measurement, a 12-month lag period was selected for PDL models. Results from these PDL models and subsequent BMA showed that 98.4% and 98.2% of the posterior probability corresponded to the zero-order model (equivalent to the moving-average) for PM_2.5_ and PM_10_, respectively (Table S1). The next most influential model was the first-order, or linear decay model, which contributed only 1.6 and 1.8% for PM_2.5_ and PM_10_, respectively. Weighted coefficients from BMA of these six models, for both PM_2.5_ and PM_10_ did not indicate substantial non-linearity or even linear decay in the response to lagged exposures over the prior 12 months (Figure S1).

## Discussion

In our study population, significant inverse associations were observed between long-term residential PM_2.5_ and PM_10_ levels and endothelial function measured by brachial artery FMD. Though associations were suggestive of stronger PM_2.5_ and PM_10_ effects in men than in women, interaction terms were non-significant. These trends persisted in age- and sex-stratified analyses, for which associations between PM_2.5_ and PM_10_ and FMD were stronger in men than in women, for both those < 50 years and greater. One explanation for stronger associations of PM_2.5_ and PM_10_ with FMD in men as compared to women is that Amish men have higher levels of physical activity than Amish women, as we have previously shown using 7-day accelerometer counts [23]. If this physical activity occurs largely outdoors, it is plausible that their personal exposure would be more highly correlated with ambient PM levels. Another explanation is that Amish women are non-smokers and have less outdoor activity. Finally, our results suggest greater effects of PM_2.5_ and PM_10_ on FMD among older adults (> 50 years), which may indicate increased susceptibility. However, because these interactions were not statistically significant, this conclusion requires replication in a larger sample size.

In contrast to FMD, a stronger association between PM_10_ and response to shear stress was observed among women compared to men, suggesting greater nitric oxide bioavailability among women.

For both PM_2.5_ and PM_10_, the data indicated selection of a 12-month averaging period was appropriate. Earlier or later exposures to PM_2.5_ or PM_10_ within the prior 12-month period did not substantially affect FMD responses, as evidenced by the overwhelming dominance of the zero-order PDL models for both PM_2.5_ and PM_10_. Results indicated stronger associations with PM_10_ compared to PM_2.5_ for equivalent averaging periods, except in the women only group for which associations were non-significant (Table 2). One possible explanation is compositional differences in PM_10_ vs. PM_2.5_. Another is that we measured endothelial function in the brachial artery, which is a relatively large blood vessel, whereas PM_2.5_ may exert the majority of its influence on smaller vasculature. Finally, the spatial misalignment of the PM_2.5_ and PM_10_ monitors may have contributed to exposure errors. The largest source of these errors appears primarily due to the apparent underestimation of PM_10_ levels at a PM_10_ monitor near to many of the participant residences; in contrast PM_2.5_ monitors were farther away and reported higher levels. We note that PM_2.5_ accounts for the majority of the mass of PM_10_ in most areas of the US [28]. Despite the limitation imposed by this spatial error, we believe the estimates of association for PM_2.5_ and PM_10_ in our study are valid because consistent, systematic overestimation of measured PM_2.5_ levels is not expected to change the rank-ordering of PM_2.5_ exposure values; similarly, for underestimation of PM_10_. However, dose-response interpretations based on this analysis should be viewed with caution. Future research on PM effects on endothelial function using personal exposure monitoring for PM_2.5_ and PM_10_ to further clarify differences in toxicity based on particle size fraction and/or composition is warranted.

Compared to previous studies, our results for PM_2.5_ are consistent in direction but larger in magnitude of effect (Table S3). One explanation is that our exposure assessment approach better described gradients in exposure resulting in less misclassification. Additionally, differences in time-activity patterns, with Amish men spending more time outside than participants in other studies, may have affected our results. Wilker et al. [34] conducted an analysis of long-term PM_2.5_ exposure and FMD among participants of the Framingham Offspring and Third Generation Cohorts (*n* = 5112) and reported a smaller effect size compared to ours (over an approximated 10 μg m^− 3^ increment: (10/1.99)*(− 0.16) = − 0.8% in FMD vs. our result of − 4.0% (Table S3)). In Krishnan et al. [13], an analysis among members of the Multi-Ethnic Study of Atherosclerosis and Air Pollution study (*n* = 3040), authors also reported an effect size for long-term PM_2.5_ and FMD smaller than the present analysis (over an approximated 10 μg m^− 3^ increment: (10/3.0)*(− 0.3) = − 1.0% in FMD vs. our result of − 4.0%). Both of these studies used average PM_2.5_ levels over one calendar-year as surrogates of long-term exposure, and thus did not average over the specific 12-months prior to FMD measurement (*i.e.,* over a “moving-window”). As stated above, our analysis supports the use of a 12-month moving-average period, when available, with regard to studies of health effects of PM_2.5_ and PM_10_ on FMD.

Although the underlying biological mechanism of these effects is not currently well understood, it has been postulated that exposure to PM_2.5_ may regulate endothelial function through altered expression or function of the enzyme nitric oxide synthase which results in reduced bioavailability of endothelium-derived nitric oxide, a key component of vascular homeostasis [22]. Recent animal studies have elucidated the role of endothelial progenitor cells in this process [8]. Our results generally showed stronger, albeit not significantly, associations of PM_10_ exposure with response to shear stress in women than in men, which is consistent with greater resiliency of women to PM-induced endothelial dysfunction, perhaps attributable to protective hormonal effects or absence of smoking in women.

Our study has several strengths. The homogeneity of the study population with regard to lifestyle and behavioral factors such as physical activity, diet, and formal education reduces the possibility that observed PM air pollution-FMD relationships are confounded by these factors. To our knowledge, this is the first study of air pollution and endothelial function in a genetically homogenous, rural population of clinically healthy individuals. Moreover, FMD was measured by a single well-trained sonographer and data was obtained by a single cardiologist using high quality-control standards. In addition, we used GIS-based spatio-temporal exposure models to predict time-varying (*i.e.,* monthly) PM_2.5_ and PM_10_ exposure estimates specific to each participant’s residential address and used this data to calculate exposure metrics based on the FMD exam date.

Limitations of our study include a lack of racial/ethnic variability such that our findings are not generalizable to other ethnicities. Also, our exposure estimates were found to contain exposure error such that PM_2.5_ estimates were sometimes slightly higher than PM_10_ estimates, limiting conclusions regarding the relative toxicity of PM_2.5_ as compared to PM_10_ from these data. Moreover, exposures to PM_2.5_ and PM_10_ were based on monthly averages, preventing our analyses from addressing acute exposures to PM_2.5_ or PM_10_.

This study provides support for associations between long-term exposure to both PM_2.5_ and PM_10_ with brachial artery FMD. This effect manifested maximally over the time course of approximately 12 months for both PM_2.5_ and PM_10_. Associations with FMD were stronger (*i.e.,* more negative) for PM_10_ than for PM_2.5_. Results were suggestive of stronger associations in men than women, though these interactions did not reach statistical significance. The findings bolster the existing evidence regarding the effects of PM air pollution on CVD risk and suggest long-term exposures to PM_2.5_ and PM_10_ are plausible early risk factors of cardiovascular events.

## Supplementary information

**Additional file 1 : Table S1.** Polynomial distributed lag (PDL) model posterior probabilities obtained from Bayesian model averaging (BMA) for zero-order through 5th order models using 12 lag periods, for PM_2.5_ and PM_10_. **Table S2.** Associations of PM_2.5_ and PM_10_ exposure metrics and FMD stratified by age (less than 50 years vs. 50 years and older) and sex for increases in normalized PM_2.5_ and PM_10_ exposure in fully adjusted models. **Table S3.** Associations of PM_2.5_ and PM_10_ exposure metrics and FMD across all participants and by sex for a 10 μg m^− 3^ increment in PM_2.5_ and PM_10_ exposure in fully adjusted models. **Figure S1.** Effect estimates from the six PDL models (zero-order through 5th order) weighted using BMA as discussed in main text, for: A) PM_2.5_ and B) PM_10_. The month prior to FMD measurement (lag zero) and 11 months prior are shown, as well as the cumulative effect over all 12 lag periods.

## Data Availability

Supporting data is not available due to confidentiality constraints involved with human subject’s research.

## Abbreviations

BMA: Bayesian model averaging
FMD: Flow-mediated dilation
PM_2.5_: Particulate matter < 2.5 μm
PM_10_: Particulate matter < 10 μm
