# Supplementary material for: Long-term exposure to particulate air pollution and brachial artery flow-mediated dilation in the Old Order Amish
Source: Environ Health. 2020 May 14;19:50. doi: 10.1186/s12940-020-00593-y (PMC7222318; doi:10.1186/s12940-020-00593-y)
Supplement: Supplementary file 1 — Additional file 1 : Table S1. Polynomial distributed lag (PDL) model posterior probabilities obtained from Bayesian model averaging (BMA) for zero-order through 5th order models using 12 lag periods, for PM2.5 and PM10. Table S2. Associations of PM2.5 and PM10 exposure metrics and FMD stratified by age (less than 50 years vs. 50 years and older) and sex for increases in normalized PM2.5 and PM10 exposure in fully adjusted models. Table S3. Associations of PM2.5 and PM10 exposure metrics and FMD across all participants and by sex for a 10 μg m− 3 increment in PM2.5 and PM10 exposure in fully adjusted models. Figure S1. Effect estimates from the six PDL models (zero-order through 5th order) weighted using BMA as discussed in main text, for: A) PM2.5 and B) PM10. The month prior to FMD measurement (lag zero) and 11 months prior are shown, as well as the cumulative effect over all 12 lag periods. [file 12940_2020_593_MOESM1_ESM.pdf]

Supplemental Material

Table S1. Polynomial distributed lag (PDL) model posterior probabilities obtained from Bayesian model averaging (BMA) for zero-order through 5th order models using 12 lag periods, for $PM_{2.5}$ and PM_{10}.

Pollutant	PDL model order (for 12 lag periods)	Posterior probability
$PM_{2.5}$	0	98.4%
	1	1.6%
	2	0.0%
	3	0.0%
	4	0.0%
	5	0.0%
PM_{10}	0	98.2%
	1	1.8%
	2	0.0%
	3	0.0%
	4	0.0%
	5	0.0%

Table S2. Associations of $PM_{2.5}$ and PM_{10} exposure metrics and FMD stratified by age (less than 50 years *vs.* 50 years and older) and sex for increases in normalized $PM_{2.5}$ and PM_{10} exposure in fully adjusted models.

Age younger than 50 years								
	Men				*Women*			
Particulate air pollution metrics (normailzed)	β	SE	*p*-value	95% CI	β	SE	*p*-value	95% CI
$PM_{2.5}$ 12-month moving-average	-0.07	0.05	0.1	-0.17, 0.03	-0.02	0.08	0.7	-0.17, 0.14
PM_{10} 12-month moving-average	-0.16	0.05	0.005	-0.25, -0.04	-0.01	0.07	0.8	-0.14, 0.12
**Age 50 years and older**								
$PM_{2.5}$ 12-month moving-average	-0.13	0.09	0.1	-0.30, 0.04	-0.07	0.07	0.3	-0.20, 0.06
PM_{10} 12-month moving-average	-0.17	0.09	0.08	-0.36, 0.02	-0.06	0.07	0.4	-0.18, 0.08

Table S3. Associations of $PM_{2.5}$ and PM_{10} exposure metrics and FMD across all participants and by sex for a 10 μg m^{-3} increment in $PM_{2.5}$ and PM_{10} exposure in fully adjusted models.

Particulate air pollution metrics (per 10 μg m^{-3})	*Across all*				*Men*				*Women*			
	β	SE	*p*-value	95% CI	β	SE	*p*-value	95% CI	β	SE	*p*-value	95% CI
$PM_{2.5}$ 12-month moving-average	-4.0	1.8	0.03	-7.5, -0.5	-4.9	2.4	0.04	-9.6, -0.3	-3.7	3.0	0.2	-9.5, 2.1
PM_{10} 12-month moving-average	-4.7	1.7	0.007	-8.2, -1.3	-7.6	2.3	0.001	-12.2, -3.0	-2.8	2.6	0.3	-7.9, 2.4

*All models adjusted for age, sex, age by sex interaction, smoking (except in models for women only because there were no ever smokers), BMI, season, year, hypertension, and base brachial artery diameter.

Figure S1. Effect estimates from the six PDL models (zero-order through 5th order) weighted using BMA as discussed in main text, for: A) $PM_{2.5}$ and B) PM_{10}. The month prior to FMD measurement (lag zero) and eleven months prior are shown, as well as the cumulative effect over all 12 lag periods.

A)

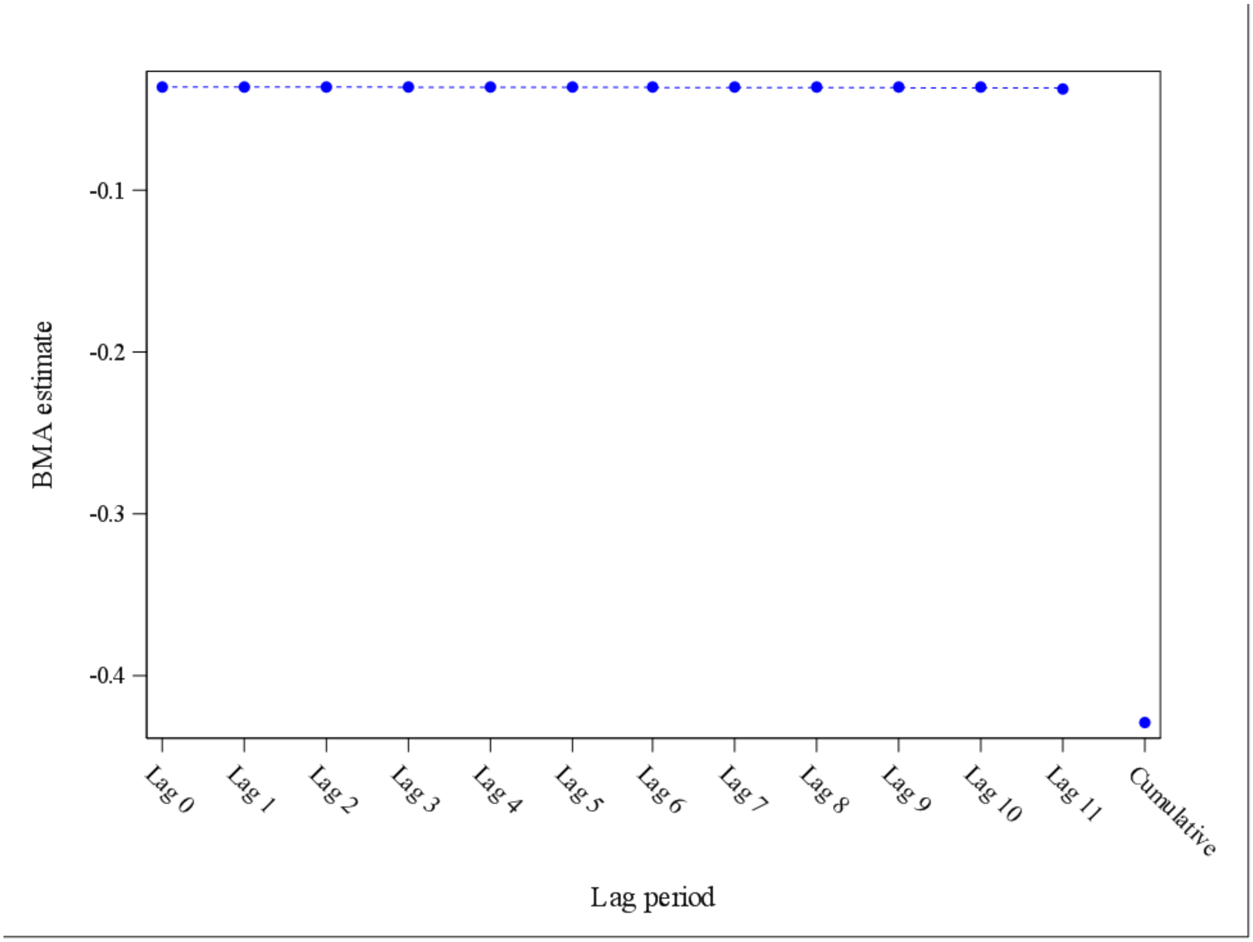

B)

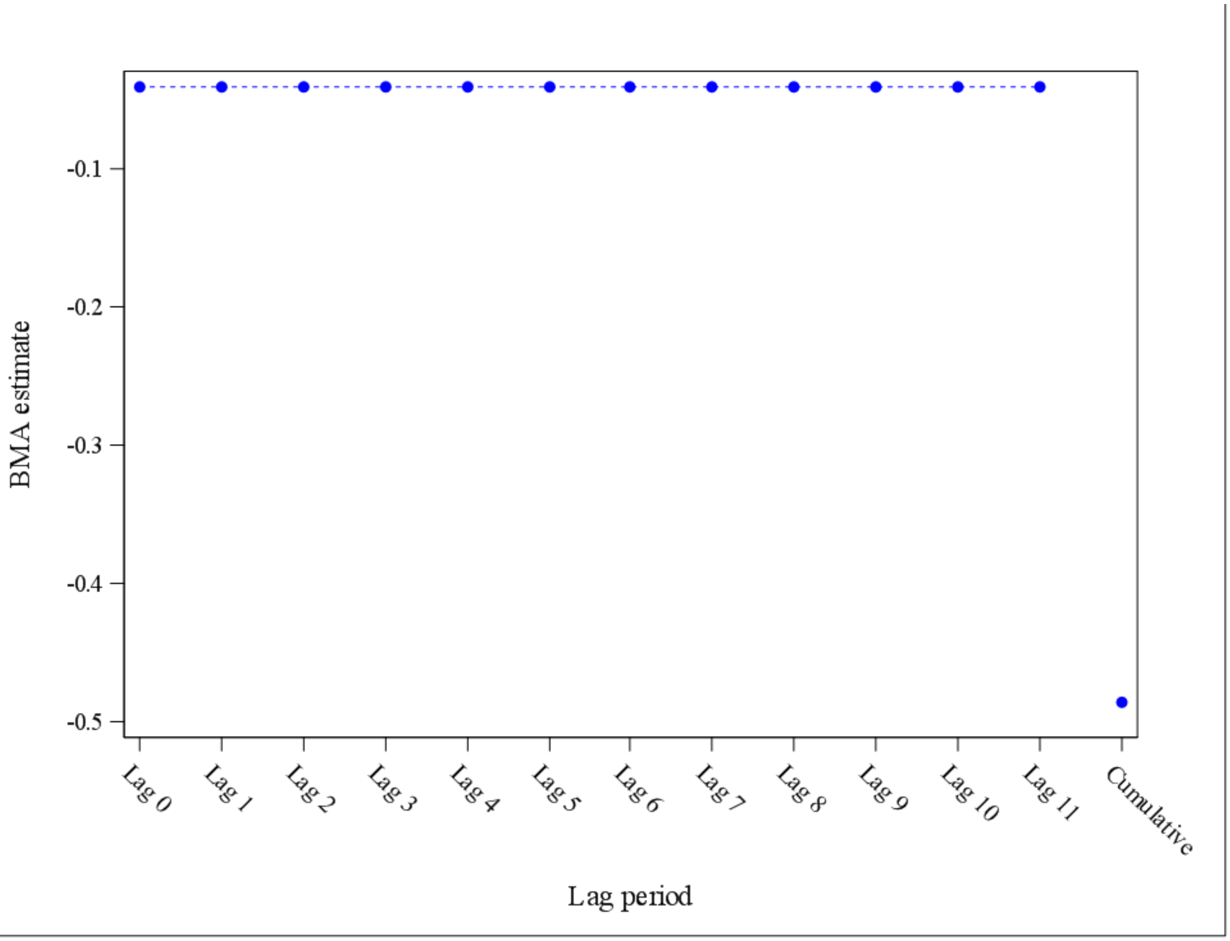
BMA estimate
-0.1
-0.2
-0.3
-0.4
-0.5
Lag 0
Lag 1
Lag 2
Lag 3
Lag 4
Lag 5
Lag 6
Lag 7
Lag 8
Lag 9
Lag 10
Lag 11
Cumulative
Lag period
